# Beyond Paralogs: The Multiple Layers of Redundancy in Bacterial Pathogenesis

**DOI:** 10.3389/fcimb.2017.00467

**Published:** 2017-11-15

**Authors:** Soma Ghosh, Tamara J. O'Connor

**Affiliations:** Department of Biological Chemistry, Johns Hopkins University School of Medicine, Baltimore, MD, United States

**Keywords:** redundancy, pathogenesis, effector, functional redundancy, genetic redundancy, *Legionella*

## Abstract

Redundancy has been referred to as a state of no longer being needed or useful. Microbiologists often theorize that the only case of true redundancy in a haploid organism would be a recent gene duplication event, prior to divergence through selective pressure. However, a growing number of examples exist where an organism encodes two genes that appear to perform the same function. For example, many pathogens translocate multiple effector proteins into hosts. While disruption of individual effector genes does not result in a discernable phenotype, deleting genes in combination impairs pathogenesis: this has been described as redundancy. In many cases, this apparent redundancy could be due to limitations of laboratory models of pathogenesis that do not fully recapitulate the disease process. Alternatively, it is possible that the selective advantage achieved by this perceived redundancy is too subtle to be measured in the laboratory. Moreover, there are numerous possibilities for different types of redundancy. The most common and recognized form of redundancy is functional redundancy whereby two proteins have similar biochemical activities and substrate specificities allowing each one to compensate in the absence of the other. However, redundancy can also exist between seemingly unrelated proteins that manipulate the same or complementary host cell pathways. In this article, we outline 5 types of redundancy in pathogenesis: molecular, target, pathway, cellular process, and system redundancy that incorporate the biochemical activities, the host target specificities and the impact of effector function on the pathways and cellular process they modulate. For each type of redundancy, we provide examples from *Legionella* pathogenesis as this organism employs over 300 secreted virulence proteins and loss of individual proteins rarely impacts intracellular growth. We also discuss selective pressures that drive the maintenance of redundant mechanisms, the current methods used to resolve redundancy and features that distinguish between redundant and non-redundant virulence mechanisms.

## Redundancy—biology's contingency plan

Bacteria are one of nature's ultimate survivalists, able to adapt to extreme and dynamic environmental conditions. One of the reasons for their robustness is redundancy, contingency plans for a given process that enhances their fitness. Genetic redundancy describes two copies of the same gene whereby the protein encoded by one can function in place of the other. A classic example of genetic redundancy occurs in metabolism, where two genes encode proteins that catalyze the same reaction (Toda et al., 1987). However, redundancy extends beyond gene duplication. Two proteins or sets of proteins with different catalytic activities can generate the same product (Wagner, 2000). The ability to synthesize a molecule *de novo* and the ability to acquire that molecule from the environment is also a form redundancy. In this case, the proteins and their functions are completely unrelated but they serve a common goal. Thus, redundancy can occur at multiple levels within a system and is largely defined by what a bacterium is trying to accomplish.

## Redundancy in microbial pathogenesis

Koch's postulates outline a set of criteria to define causal relationships between pathogens and disease (Koch, 1891; Evans, 1976). With advances in molecular biology techniques and bacterial genetics, Stanley Falkow proposed a molecular version of Koch's postulates to define virulence factors responsible for the pathogenesis of an individual microorganism (Falkow, 1988). The postulate sets an exclusive condition where disruption of a gene should result in a virulence defect and that phenotype should be reversed upon allelic replacement of the gene. For decades, the postulate has been used to identify many virulence factors in numerous pathogens (Isberg et al., 1987; Hersh et al., 1999). At the same time however, a growing number of genes that failed Falkow's criteria but played important roles in disease began to emerge (Falkow, 2004; Choy et al., 2012; Gaspar and Machner, 2014). The lack of phenotypes associated with genetic mutations was attributed to redundancy amongst virulence factors. While redundancy is not the only explanation for this phenomenon (discussed below), it is becoming a common feature in microbial pathogenesis with examples from *Legionella* (Luo and Isberg, 2004; Belyi et al., 2006), *Pseudomonas* (Kvitko et al., 2009; Cunnac et al., 2011), *Yersinia* (Ratner et al., 2016), *Chlamydia* (Cocchiaro and Valdivia, 2009), *Salmonella* (Zhou et al., 2001), and *Mycobacterium* (Downing et al., 2005; Ganapathy et al., 2015). While an exciting challenge for microbiologists, redundancy is a major obstacle in identifying virulence factors, deciphering their roles in disease and developing new therapeutic agents to combat infection.

## Redundancy in *Legionella* pathogenesis

*Legionella pneumophila* is an intracellular bacterial pathogen with a broad host range spanning over 15 species of amoebae and ciliated protozoa (Rowbotham, 1980) to mammalian macrophages (Horwitz and Silverstein, 1980). Intracellular growth of *L. pneumophila* requires a number of key events be accomplished. *L. pneumophila* must disrupt endocytic and autophagic targeting of its membrane-bound compartment, termed the *Legionella*-containing vacuole (LCV) to avoid digestion in the lysosome (Horwitz, 1983; Berger et al., 1994; Swanson and Isberg, 1995; Wiater et al., 1998; Choy et al., 2012); transform the phagosome into a replication-permissive compartment (Kagan and Roy, 2002; Derre and Isberg, 2004; Kagan et al., 2004); acquire nutrients to grow (Sauer et al., 2005; Allard et al., 2009; Isaac et al., 2015); expand and maintain the integrity of the replication vacuole to accommodate increasing bacterial numbers (Laguna et al., 2006; Creasey and Isberg, 2012); avoid detection by host innate immune recognition (Laguna et al., 2006; Zamboni et al., 2006; Coers et al., 2007; Fontana et al., 2011; Pereira et al., 2011; Creasey and Isberg, 2012; Barry et al., 2013); inhibit host cell death to maintain an intracellular environment that supports replication (Losick and Isberg, 2006; Abu-Zant et al., 2007); and eventually, exit from the host cell (Horwitz and Silverstein, 1980). As it turns out, *L*. *pneumophila* employs multiple strategies to accomplish each of these tasks.

With Falkow's molecular Koch's postulates in mind, several genetic screens to correlate gene disruptions with virulence defects have been employed to identify *L*. *pneumophila* virulence genes (Berger and Isberg, 1993; Sadosky et al., 1993; VanRheenen et al., 2004; Laguna et al., 2006). Parallel genetic screens independently identified a collection of 26 genes encoding components of a Type IVb secretion system, subsequently named Icm/Dot (Marra et al., 1992; Berger and Isberg, 1993; Brand et al., 1994). Mutations in *icm*/*dot* genes abolish *L. pneumophila* intracellular growth in macrophages (Berger and Isberg, 1993; Brand et al., 1994) and amoebal hosts (Segal and Shuman, 1999) demonstrating a critical role for the Icm/Dot complex in *L. pneumophila* pathogenesis. The identification of Icm/Dot was not surprising as numerous pathogens employ secretion systems to deploy proteins, termed effectors to the host cell to establish growth. Yet the search for Icm/Dot translocated substrates (IDTS) using similar genetic screening strategies was relatively unsuccessful, identifying only a small handful of IDTS-encoding genes that were important for *L. pneumophila* pathogenesis (VanRheenen et al., 2004; Laguna et al., 2006; Isaac et al., 2015). As a consequence, more creative genetic screening strategies were implemented (Luo and Isberg, 2004; Campodonico et al., 2005): not only did this lead to the identification of the first set of IDTS but also the presence of multiple paralogs of many IDTS in the *L. pneumophila* genome (Luo and Isberg, 2004). As a result, the lack of phenotypes associated with genetic mutations in a single IDTS was attributed to redundancy.

The presence of multiple IDTS paralogs was the first evidence of redundancy in *L. pneumophila* pathogenesis. However, the simultaneous deletion of all paralogs from a single family of IDTS did not impair *L. pneumophila* intracellular growth (Bardill et al., 2005). The simplest explanation was that these genes were dispensable under the experimental conditions tested. However, the subsequent use of biochemical and bioinformatics-based approaches had begun to define a collection of 270 translocated proteins (de Felipe et al., 2005; Huang et al., 2011; Zhu et al., 2011). In the process, pairs of IDTS that modulate the same host protein via different mechanisms or different components of the same pathway were identified (Nagai et al., 2002; Machner and Isberg, 2006; Murata et al., 2006; Belyi et al., 2008). In parallel, genetic screens in host cells to identify host factors important for *L. pneumophila* pathogenesis demonstrated that while depletion of a single host factor rarely impaired *L. pneumophila* replication, the combined deletion of pairs of host proteins that function in common processes significantly disrupted intracellular growth of the bacterium (Dorer et al., 2006). Collectively, these results suggested that redundancy extends beyond paralogs to more complex mechanisms that function at pathway and system levels, providing an explanation for the lack of phenotypes for mutants lacking all members of a paralogous family of IDTS. In support of this, it was subsequently shown that deleting specific combinations of unrelated IDTS impairs *L. pneumophila* intracellular growth while deletion of each gene individually failed to elicit a phenotype (O'Connor et al., 2011, 2012). Thus, redundancy appeared to be a multi-tiered phenomenon integrating many different forms.

## Types of redundancy

**Figure 1:**
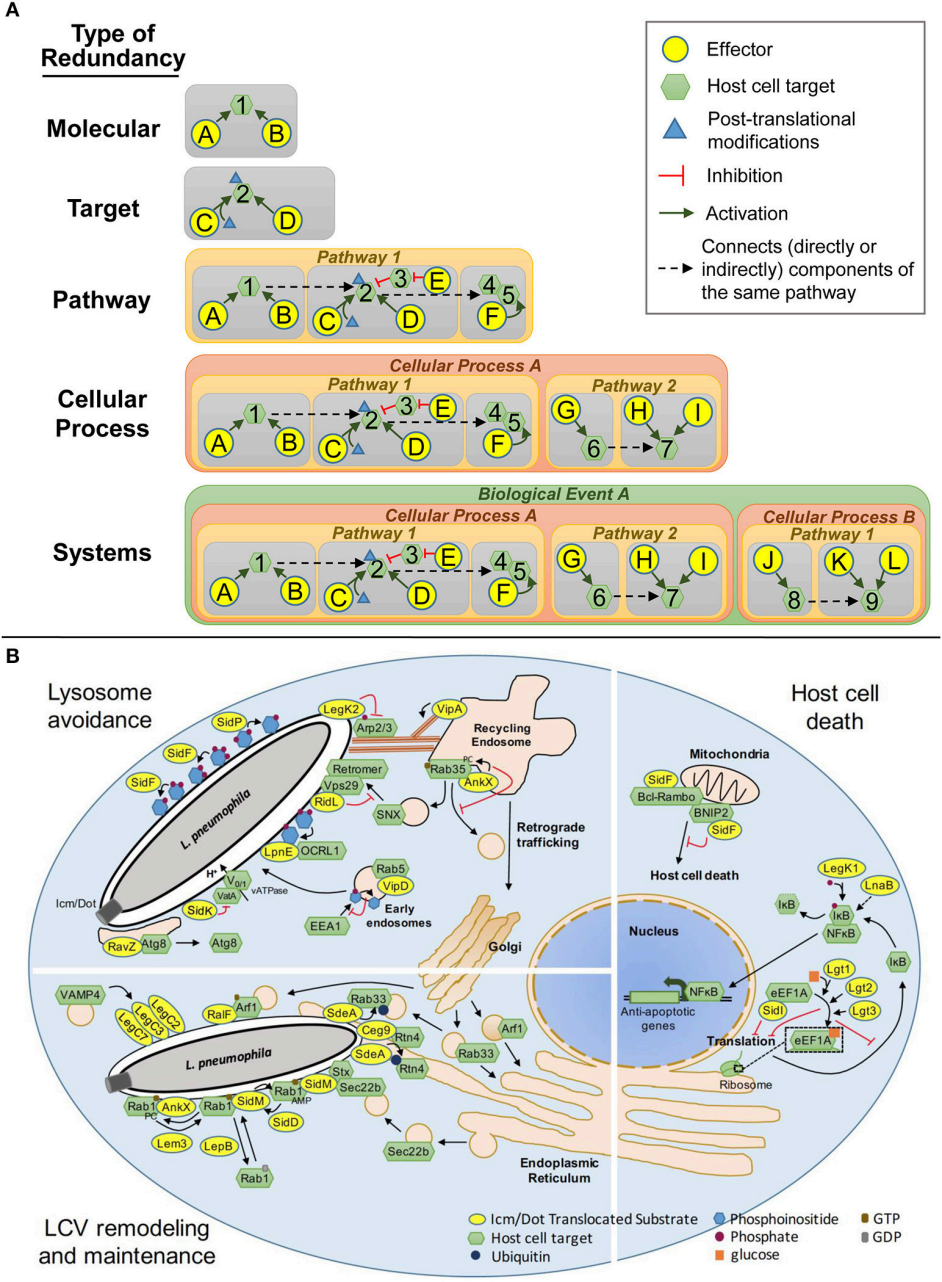
Types of Redundancy. **(A)** Schematic representations of the 5 classes of redundancy: *Molecular*, two or more effectors that modify the same host target using the same molecular mechanism; *Target*, effectors that modulate the same host protein using different molecular mechanisms; *Pathway*, effectors that modulate a single host pathway but target different components of that pathway; *Cellular Process*, effectors that target redundant or complementary host pathways that collectively govern a single cellular process; *System*, effectors that modulate more than one host cellular process to accomplish a common goal. **(B)** Redundant Icm/Dot translocated substrates that modulate lysosomal trafficking, vacuole remodeling and maintenance and host cell death in *Legionella* pathogenesis.

Redundancy in microbial pathogenesis manifests in many forms that encompass a broad spectrum of functional relationships and multiple levels of biological systems: this complexity necessitates a structured nomenclature to define the different types of redundancy. Genetic and functional redundancy are often used interchangeably, defining compensatory roles for two proteins with the same biochemical activities that allow one to substitute in place of the other. However, the use of function can be somewhat subjective, as it can refer to a precise biochemical activity or more generally, to the impact of that activity on a particular pathway or cellular process. As a consequence, the term functional redundancy has been omitted here as it could be used to describe more than one type of redundancy outlined below. Instead, we propose 5 types of redundancy (Figure 1A): molecular, target, pathway, cellular process, and system redundancy that incorporate the biochemical activities of effectors, their host target specificities, their impact on host cell biology and their contributions to pathogenesis. In many cases, virulence strategies are multi-tiered encompassing several types of redundancy.

### Molecular redundancy

Molecular redundancy defines two or more effectors that modify the same host target using the same molecular mechanism (Figure 1A). In this case, one effector can function in place of the other because it has the same activity and target specificity as its counterpart(s). In some cases, molecular redundancy is likely to be a byproduct of gene duplication however, this is not the sole source with examples of horizontal gene transfer and convergent evolution leading to the presence of molecularly redundant proteins.

Molecular redundancy is exemplified by the *L. pneumophila* SidE family of IDTS, SidE, SdeA, SdeB, and SdeC (Luo and Isberg, 2004). Individual paralogs consist of a mono-ADP-ribosyltransferase domain, a deubiquitylation domain, and a phosphohydrolase domain that collectively catalyze the ubiquitination of the host proteins Reticulon 4 (Rtn4) (Kotewicz et al., 2016) and Rab33b (Qiu et al., 2016) (Figure 1B). While each member of this family is individually dispensable for intracellular replication, the simultaneous deletion of all four members impairs growth of *L. pneumophila* in the amoebal hosts *Acanthamoebae castellanii* (Bardill et al., 2005) and *Dictyostelium discoideum* (Qiu et al., 2016). The virulence defects can be rescued by SdeA alone (Bardill et al., 2005; Qiu et al., 2016) demonstrating that, at least in these two hosts, a single paralog is sufficient for *L. pneumophila* intracellular replication.

A second example of molecular redundancy in *L*. *pneumophila* is the Lgt family of proteins consisting of Lgt1, Lgt2/LegC8, and Lgt3/LegC5 (de Felipe et al., 2005; Belyi et al., 2006) (Figure 1B). Each paralog is a functional glucosyltransferase that covalently modifies the host protein elongation factor 1A (eEF1A) at serine 53 via mono-O-glycosylation (Belyi et al., 2006, 2008). Modification of eEF1A by any of the three paralogs impairs host protein synthesis (Belyi et al., 2006, 2008). The simultaneous deletion of all three 3 paralogs does not impair *L. pneumophila* intracellular growth nor does it completely abolish host protein translation in infected cells (Belyi et al., 2006, 2008) suggesting that *L. pneumophila* encodes additional IDTS that modulate this process.

#### Effectors with similar activities can be misinterpreted as redundant

Effector paralogs are most readily identified by sequence and/or structural similarities. However, homology does not necessarily indicate that two proteins perform the same function and therefore have molecular redundancy. For example, the IDTS VipD is targeted to early endosomes through its interaction with the host protein Rab5 (Gaspar and Machner, 2014). Binding to Rab5 activates VipD phospholipase activity resulting in dephosphorylation of phosphoinositol 3-phosphate on endosomes (Gaspar and Machner, 2014). Sequence homology comparisons identified three paralogs of VipD encoded in the *L. pneumophila* genome: VdpA, VpdB, and VpdC, each consisting of a functional phospholipase domain based on the conservation of all active site and catalytic residues (VanRheenen et al., 2006; Gaspar and Machner, 2014). However, *in vitro* binding assays demonstrated that unlike VipD, neither VpdA nor VpdB bind Rab5 (Gaspar and Machner, 2014). Thus, while it appears that the catalytic activities of these four proteins are conserved and they are all likely to alter host phosphoinositide pools, their respective binding partners and the host pathways they modulate may vary significantly.

The unlikelihood of redundancy amongst larger families of effectors with conserved activities or domains is more apparent, as demonstrated by the five F-box domain-containing proteins of the E3 ubiquitin ligase family in *L. pneumophila* (Ensminger and Isberg, 2010). The F-box protein provides substrate specificity to the E3 ubiquitin ligase complex, typically Skp-Cullin-F-box (SCF) (Zheng et al., 2002). Despite their common F-box domain, only LegU1, LegAU13/AnkB, and LicA interact with Skp1 while only LegU1 and LegAU13/AnkB interact with CUL1 (Ensminger and Isberg, 2010). Moreover, the pattern of host protein ubiquitination varies significantly between the five IDTS (Ensminger and Isberg, 2010). Thus, despite similar biochemical activities, the host proteins they target for ubiquitination and the corresponding host processes they impact are likely to differ. Indeed, many families of IDTS with common functional domains such as kinases, phosphatases, ankyrin-repeat, or coil-coil domains (de Felipe et al., 2005) typically have additional, unrelated functional domains that set them apart.

Effectors that exhibit similar activities or target specificities *in vitro* or *in vivo* outside the context of an infection can also be misinterpreted as redundant, as these similarities may not translate to redundancy in the context of a host under native conditions. Additionally, enzymatic functions and/or target specificities defined *in vitro* could be biased based on the substrates and/or assays used to investigate them and thus, misleading as to their true functions within a host. As a consequence, *in vitro* studies may suggest redundancy between two proteins that are in fact quite distinct in the context of an infection.

### Target redundancy

Target redundancy defines effectors that modulate the same host protein using different molecular mechanisms (Figure 1A). In this case, the activity of one effector cannot replace the other but can have a similar impact on the function of the targeted host protein, and its component pathway. Thus, contrary to molecular redundancy, target redundancy defines redundant strategies rather than redundant activities. Effectors that are redundant at the target level are more difficult to identify because they typically lack sequence, structural and functional similarity.

Target redundancy is exemplified by the IDTS SidM/DrrA (Machner and Isberg, 2006; Murata et al., 2006) and AnkX/LegA8/AnkN (Pan et al., 2008; Mukherjee et al., 2011; Allgood et al., 2017), herein after referred to as AnkX (Figure 1B). Both effectors modulate the activity of the host small GTPase Rab1 to remodel the LCV but do so by different molecular mechanisms. SidM/DrrA AMPylates the GTP-bound form of Rab1 preventing GTP to GDP exchange by its cognate GAP protein (Muller et al., 2010), whereas AnkX phosphocholinates GTP-bound Rab1, locking it in the active state (Mukherjee et al., 2011). Two additional effectors SidD and Lem3 reverse Rab1 constitutive activation by de-AMPylation and de-phosphocholination, respectively (Neunuebel et al., 2011; Tan and Luo, 2011). Thus, *L. pneumophila* encodes two sets of IDTS, SidM-SidD and AnkX-Lem3 that are both able to regulate Rab1 activity but do so through different mechanisms.

A second example of target redundancy is observed between the glucosyltransferases Lgt1, Lgt2, Lgt3 (Belyi et al., 2008), and SidI (Shen et al., 2009) (Figure 1B). While the Lgt proteins and SidI both impair host protein synthesis by targeting eEFA1, SidI appears to do so by an alternative mechanism. Similar to Lgt1, Lgt2, and Lgt3, SidI directly interacts with eEFA1 to impair its function however, direct binding is not solely responsible for this effect (Shen et al., 2009). If SidI inactivates eEF1A through modification, the lack of a glycosyltransferase domain suggests it is likely to differ from glycosylation. In addition to Lgt1, Lgt2, Lgt3, and SidI, a fifth effector, SidL has been implicated in impairing host protein synthesis (Fontana et al., 2011), although the mechanism has yet to be elucidated including whether this occurs through eEF1A or another component of the translation machinery. Moreover, the deletion of all five of these IDTS only partially restores host protein synthesis (Fontana et al., 2011), suggesting that additional IDTS regulate this process. Thus, this example encompasses multiple types of redundancy from molecular and target redundancy to pathway and possibly, cellular process redundancy (see below). We predict that many virulence strategies will similarly consist of more than one type of redundancy.

#### Effectors with similar targets but different activities can be misinterpreted as redundant

It is important to distinguish between effectors that modulate the activity of common host proteins but do not achieve the same effect on the component host pathway: these types of effectors are not redundant. An example of non-redundant IDTS with a common target is SidM/DrrA and LepB. SidM/DrrA functions as a GDI displacement factor to recruit Rab1 to the *Legionella* vacuole, then constitutively activates Rab1 by locking it in the GTP bound form via covalent modification (Machner and Isberg, 2007; Muller et al., 2010). Upon de-AMPylation of Rab1 by SidD (Neunuebel et al., 2011), LepB acts as a Rab1 GTPase activating protein (GAP) promoting GTP hydrolysis and release of Rab1 from the LCV (Ingmundson et al., 2007). Although, SidM and LepB both target Rab1, they have opposite effects on its activity and distribution which differentially impacts Rab1-mediated vesicle trafficking events. Thus, SidM/DrrA and LepB are not target redundant.

### Pathway redundancy

Pathway redundancy defines effectors that modulate a single host pathway but target different components of that pathway (Figure 1A). Sets of effectors that belong to this category can manipulate different proteins in a single complex, different components at various steps along the pathway or regulators of the pathway. However, while the mechanisms and the host proteins used to modulate the pathway differ, the outcome of that modulation is the same and these effectors collectively serve to achieve a common goal.

Pathway redundancy is illustrated by the IDTS VipD and SidK that both modulate the endocytic pathway but do so by targeting different components at different stages of LCV maturation on the way to the lysosome (Figure 1B). On early endosomes, VipD dephosphorylates PI3P, which functions as an anchor for the tethering protein EEA1 (Gaspar and Machner, 2014). The lack of EEA1 at endosomal surfaces prevents endosome fusion with the LCV (Gaspar and Machner, 2014). Vacuole acidification occurs downstream of early endosome fusion events and is mediated by vATPases, multi-component proton pumps (Forgac, 2007). SidK directly binds VatA, a component of the vATPase to inhibit its function (Xu et al., 2010). While VipD can impair early endosome fusion with the LCV, it is not sufficient to avoid endosomal fusion completely as 40% of LCVs containing wild type bacteria stain positive for the early endosomal marker Rab5 (Gaspar and Machner, 2014). SidK acts as part of a contingency plan when endocytic maturation of the LCV is not completely thwarted. Moreover, while vacuoles containing the Δ*vipD* mutant are more likely to accumulate Rab5 than those containing wild type bacteria, the frequency is significantly lower than that observed for a *dot*- mutant (Gaspar and Machner, 2014): this suggests that other effectors function to modulate the endocytic pathway. Several effectors including VipA, VipF, SetA, and Ceg19 are likely candidates based on their ability to disrupt trafficking along the vacuole sorting pathway in yeast (Shohdy et al., 2005; Franco et al., 2012).

A second example of pathway redundancy is observed between the effectors RidL, LpnE, and AnkX which target separate mediators of retrograde trafficking between endosomes and the *trans*-Golgi network to alter the fate of the LCV (Figure 1B). RidL directly interacts with the retromer complex subunit Vps29 to compete with endosome sorting nexins for retromer and PI3P binding (Finsel et al., 2013). LpnE directly interacts with OCRL1 (Weber et al., 2009), a phosphoinositol 5-phosphatase that regulates retrograde trafficking by altering phosphoinositide phosphate pools. AnkX phosphocholinates Rab35 (Mukherjee et al., 2011), a regulator of cargo sorting and recycling from recycling endosomes. Modification of Rab35 prevents microtubule-dependent endosomal vesicle transport to the LCV (Pan et al., 2008). Loss of RidL, LpnE, or AnkX moderately increases the frequency of LAMP1 staining of LCVs demonstrating that the all three effectors independently contribute to disrupting maturation of the LCV along the endocytic pathway (Newton et al., 2007; Pan et al., 2008; Finsel et al., 2013).

### Cellular process redundancy

Cellular process redundancy occurs when sets of effectors compensate for one another by targeting redundant or complementary host pathways that collectively govern a single cellular process (Figure 1A). An example of a cellular process that is mediated by multiple pathways is the unfolded protein response (UPR). The UPR is activated through three separate sensory pathways: inositol requiring enzyme-1 (IRE1), protein kinase RNA-like ER kinase (PERK), and activating transcription factor 6 (ATF6) (Walter and Ron, 2011). Distinct pathways allow the cell to respond to multiple signs of ER stress enhancing the sensitivity and breadth of the sensory system but all pathways lead to a common response that includes global translation inhibition, upregulation of ER stress proteins, ER membrane expansion and under extreme conditions, activation of pro-apoptotic pathways (Walter and Ron, 2011). While multiple pathways provide robustness to the host, it affords pathogens multiple ways to hijack a cellular process and when necessary, the ability to do so without completely abolishing the cellular process, which can have negative, even detrimental effects on the pathogen itself.

A critical event in *Legionella* pathogenesis is remodeling and maintenance of the LCV to support bacterial replication: this is accomplished through the recruitment of ER-derived membrane material. Three parallel mechanisms by which *Legionella* achieves this have been described (Figure 1B). The SdeA, SdeB, and SdeC family of IDTS drives rearrangement of tubular ER and its association with the LCV through ubiquitination of Rtn4 (Kotewicz et al., 2016), a regulator of tubular ER dynamics (Zurek et al., 2011) (Figure 1B). SidM/DrrA and RalF target components of the early secretory pathway to redirect vesicles trafficking between the ER and the Golgi to the LCV. SidM/DrrA does so by recruiting and activating Rab1 at the LCV and promoting non-canonical functional pairing between the plasma membrane tSNARE syntaxins at the LCV and the ER-derived vesicle vSNARE Sec22b (Arasaki et al., 2012). RalF does so by recruiting and activating the host protein ARF1 at the LCV (Nagai et al., 2002) (Figure 1B). The loss of either SidM/DrrA or RalF alters the timing and efficiency of ER protein accumulation at the LCV (Nagai et al., 2002; Ingmundson et al., 2007) demonstrating redundant roles for these proteins in LCV remodeling.

A second example of redundancy at the level of cellular processes is SidF and SidP (Figure 1B). Each effector contributes to modulation of host lipid metabolism to modulate the relative abundance of phosphoinositides (PIs) at the LCV, specifically conversion from a PI(3)P rich environment to a PI(4)P rich environment. SidF is a phosphoinositide 3-dephosphatase with specificity for PI(3,4)P_2_ and PI(3,4,5)P_3_ preventing PI(3)P accumulation at the LCV (Banga et al., 2007) while SidP is a phosphoinositide 3-phosphatase that hydrolyzes PI(3)P and PI(3,5)P_2_ removing PI(3)P from the LCV (Toulabi et al., 2013). The host protein ORCL1, a PI(4,5)P_2_ 5-phosphatase also localizes to the LCV and thus may also promote PI(4)P accumulation at the surface (Weber et al., 2009) (Figure 1B). OCRL1 targeting to the LCV is Icm/Dot-dependent but the specific IDTS required for this has yet to be determined (Weber et al., 2009). Phosphoinositides distinguish individual organelle membranes in the host cell and serve as anchors for organelle-specific host proteins. Several IDTS exploit PIs decorating the LCV to anchor themselves to the surface. Many of these IDTS have been implicated in LCV remodeling including SidM/DrrA, SidC, LidA, and RidL (Machner and Isberg, 2006; Murata et al., 2006; Finsel et al., 2013; Hsu et al., 2014). Loss of SidF impairs Rab1 recruitment to the LCV (Toulabi et al., 2013), likely as a consequence of the inability of SidM to attach itself to the LCV surface. Thus, PI dynamics play a central role determining the repertoires of IDTS at the vacuole surface and thus the fate of the *Legionella* vacuole.

### System redundancy

System redundancy defines effectors that modulate more than one host cellular process to accomplish a single task (Figure 1A). An example in biology of a single event that is governed by multiple host cellular processes is cell death. Cell death can be achieved through apoptosis, necrosis, pyroptosis, or autophagy. Each process may be triggered by different cues and the mechanisms by which the cell is terminated may vary but the result is the same—death. In some cases, components mediating these pathways are completely distinct; in other cases they may overlap. For a pathogen, the more options at its disposal for manipulating the host cell to accomplish a specific goal, the greater the likelihood of its success. System redundancy provides yet another layer of insurance by allowing a pathogen to tap into multiple cellular processes to ensure completion of a critical event.

Intracellular growth of *L. pneumophila* requires the viability of the host cell but cell death is induced by host cells when bacteria cannot be eradicated through lysosomal targeting. *L. pneumophila* regulates host cell death by targeting host signal transduction, translation, and apoptosis (Figure 1B). The IDTS LnaB and LegK1 activate the host transcription factor, nuclear factor κB (NFκB) causing upregulation of anti-apoptotic pathway-associated genes (Losick and Isberg, 2006; Ge et al., 2009). While the mechanism of action of LnaB is unknown, *in vitro* studies suggest that LegK1 promotes the degradation of the NFκB inhibitor IκB through direct phosphorylation (Ge et al., 2009). Lgt1, Lgt2, Lgt3, SidI, and SidL promote prolonged NFκB signaling by blocking host protein synthesis and thus cellular levels of IκB from being replenished (Fontana et al., 2011). SidF promotes host cell survival by inhibiting the activity of the pro-apoptotic proteins BNIP3 and Bcl-Rambo through direct binding (Banga et al., 2007). Thus, *L*. *pneumophila* orchestrates the induction of host cell survival mechanisms while simultaneously obstructing host cell death pathways by targeting distinct cellular processes.

Lysosomal avoidance by *L*. *pneumophila* is orchestrated through four separate cellular processes: the endocytic pathway using VipD (Gaspar and Machner, 2014) and SidK (Xu et al., 2010); retrograde transport via RidL (Finsel et al., 2013), LpnE (Weber et al., 2009), and AnkX (Mukherjee et al., 2011); actin cytoskeleton dynamics through LegK2 (Michard et al., 2015) and VipA (Franco et al., 2012); and autophagy by RavZ (Choy et al., 2012) (Figure 1B). The mechanisms of action of VipD, SidK, RidL, LpnE, and AnkX have been discussed previously (see section Pathway Redundancy). Altering endosome transport to the LCV is also achieved by manipulating the actin cytoskeleton. LegK2 phosphorylates the Arp2/3 complex subunits ARPC1B and ARP3 (Michard et al., 2015): this prevents actin nucleation at the site of the LCV thus perturbing endosome trafficking to the LCV (Michard et al., 2015). In contrast, VipA localizes to endosomes and promotes actin polymerization by directly binding to actin. In yeast, VipA impairs vacuole sorting and thus is predicted to similarly alter organelle trafficking during infection (Shohdy et al., 2005; Franco et al., 2012). Host cells are not without their own forms of redundancy. When bacteria fail to be delivered to the lysosome, host cells can also target pathogens to the lysosome via autophagy (Xie and Klionsky, 2007). The IDTS RavZ localizes to the LCV where it irreversibly deconjugates the autophagic protein Atg8 thereby preventing autophagasome membrane nucleation at the site of the LCV (Choy et al., 2012).

System redundancy is also exemplified by SidM/DrrA, SdeA, SdeB, SdeC, RalF, and a functional complex formed by LegC2/YlfB, LegC3, and LegC7 (Figure 1B). SidM/DrrA, SdeABC, and RalF modulate LCV remodeling by hijacking tubular ER dynamics and vesicle trafficking along the early secretory pathway (see section Cellular Process Redundancy). LegC2/YlfB, LegC3, and LegC7 collectively mimic Q-SNARE proteins and directly bind the R-SNARE protein VAMP4 (Shi et al., 2016). LegC2/YlfB-LegC3-LegC7/YlfA complex pairing with VAMP4 diverts VAMP4-containing vesicle trafficking along the retrograde transport pathway between endosomes and the *trans*-Golgi network to the LCV (Shi et al., 2016). *L. pneumophila* mutants lacking LegC2/YlfB and LegC7/YlfA show reduced accumulation of the ER marker calnexin at the LCV but do not exhibit an increase in LAMP1 staining (Campodonico et al., 2016). Thus, recruitment of VAMP4-containing vesicles serves to remodel and maintain the LCV but does not impact LCV trafficking to the lysosome (Campodonico et al., 2016; Shi et al., 2016). Differential targeting of endosomes to the LCV suggests the existence of distinct populations of endosomal vesicles, some of which are actively recruited to the LCV to enable *L*. *pneumophila* replication while others are actively excluded because they promote *L*. *pneumophila* trafficking to the lysosome.

#### Effectors and host target specificity

Several studies have identified a number of effectors capable of interacting with more than one host target that often function in more than one host pathway or host cellular process. The most common example in *L*. *pneumophila* pathogenesis is IDTS that target host Rab proteins, the gatekeepers of membrane transport and trafficking. In addition to Rab1, SidM/DrrA also binds Rab8B, Rab10, and Rab27A (Machner and Isberg, 2006; Yu et al., 2015). Similarly, the IDTS LidA binds activated Rab1 and Rab6A (Machner and Isberg, 2006; Murata et al., 2006; Chen and Machner, 2013) but has also been shown to interact with Rab8B, Rab10, and Rab27A (Yu et al., 2015). Lpg0393 is a guanine nucleotide exchange factor for Rab5, Rab21, and Rab22, all of which are associated with endosomal trafficking (Sohn et al., 2015) while PieE can interact with Rab1, Rab2, Rab5c, Rab6a, and Rab7 (Mousnier et al., 2014) which encompass various stages of secretory, endocytic, and endosome recycling pathways as well as late endosome- and autophagosome-lysosome fusion events (Stenmark, 2009). While overlapping functions between IDTS may provide a source of redundancy and thus, insurance against failure to complete critical events in the infection cycle, it can also be a potential source of decreased specificity. In cases where *L. pneumophila* has to exploit subtle differences in host cellular pathways, for instance to discriminating between subpopulations of endosomal vesicles, redundancy may be less beneficial. Importantly, many of the Rab protein targets were identified using *in vitro* systems or *in vivo* systems outside the context of infection. In the case of SidM/DrrA, initial screening experiments identified seven putative Rab protein targets but subsequent validation experiments narrowed the list down to only two (Yu et al., 2015). Thus, extreme caution has to be exercised in assigning redundant functions before the biological relevance of effector-host target interactions is determined.

## Selective pressures driving the maintenance of redundant virulence proteins

Genetic redundancy is unstable over time. Genes performing similar functions tend to experience genetic drift, unless each gene undergoes independent selective pressure (Clark, 1994; Force et al., 1999; Bergthorsson et al., 2007). So how are redundant proteins maintained? The simplest explanation is that so-called redundant effectors have both overlapping and distinct functions and that selection for their independent activities drives the maintenance of their redundant functions. For example, within the Lgt1/Lgt2/Lgt3/SidI/SidL family of IDTS that inhibit protein synthesis by targeting eEF1A, SidI also interacts with eEF1Bγ (Shen et al., 2009) another component of the translation machinery (Browne and Proud, 2002). Similarly, Lgt1 has a second putative binding partner, Hsb1 that plays a role in mRNA surveillance during translation (Belyi et al., 2009). In addition, members of this family vary in their ability to block the unfolded protein response during *L*. *pneumophila* infection (Hempstead and Isberg, 2015; Treacy-Abarca and Mukherjee, 2015). While the significance of these differences has not been elucidated, the independent activities of individual members of this group may be responsible for their maintenance in the genome despite their apparent redundant functions.

Redundancy amongst effectors may compensate for temporal or regulatory differences in gene expression. For instance, Lgt1 is expressed early in the infection cycle while Lgt3 is expressed at later stages prior to bacterial egress (Belyi et al., 2008). The overlapping functions of effectors may allow a specific host processes to be modulated throughout the infection cycle despite differences in their individual expression patterns. Differences in gene expression between redundant effectors may correlate with requirements for their non-overlapping functions at different stages of the infection cycle or differences in the regulatory mechanisms controlling their expression. While redundancy resulting from gene duplication is likely to establish common regulatory networks for individual paralogs, this is unlikely for independently acquired redundant effector genes that are dispersed throughout the genome. Indeed, the mechanisms by which newly acquired effector genes are integrated into existing regulatory networks are not well established. Conservation of redundant effectors may ensure their functions are fulfilled despite variations in their respective gene expression patterns.

Redundancy between effectors may drive the maintenance of redundant virulence strategies when a host protein, pathway or process is impaired by a single effector but not completely abolished. For instance, while VipD can impair fusion of early endosomes with the LCV, it is not sufficient to avoid it completely (Gaspar and Machner, 2014). Variations in the numbers of endosomes in a host cell, the timing and amount of VipD translocated into the host cell, the efficiency of VipD targeting to endosomes, variations in substrate abundance and/or rates of catalysis or the efficiency of endosome fusion with the LCV may render VipD insufficient to avoid downstream events of the endocytic pathway. SidK (Xu et al., 2010) is part of a back-up plan when inhibition of endosome fusion with the LCV is incomplete. As many IDTS, including VipD are toxic when expressed at high levels, the need to limit effector abundance may restrict the ability of any one effector to completely control a particular event. Additional IDTS like LegK2 (Michard et al., 2015), AnkX (Mukherjee et al., 2011), RidL (Finsel et al., 2013), and VipA (Franco et al., 2012) allow *L. pneumophila* to impair endocytic maturation of the LCV at different points without obliterating major cellular processes.

Redundancy can provide an advantage when enhanced fidelity is required for critical functions (Thomas, 1993). Variations in the host cell type or fluctuations in their external environment may necessitate redundant virulence strategies. In its natural habitat, *L*. *pneumophila* is destined to encounter many amoebal species thus, the greater the number of amoebae *L. pneumophila* can survive and replicate within, the greater its fitness. The importance of individual IDTS could be impacted by multiple factors: differences in amoebal cell biology, nutrient availability, variations in host targets that impact their recognition or manipulation by IDTS or differences in the components or pathways governing cellular processes targeted by *L*. *pneumophila*. Maintaining a large cohort of IDTS arms the bacterium with the specific combinations of IDTS necessary for optimal growth in multiple hosts but as a consequence may indirectly result in the accumulation of IDTS that perform overlapping or redundant functions under certain circumstances. Redundancy may also provide a means for pathogens to evolve virulence strategies without compromising fitness. For pathogens that are subject to dynamic and unpredictable environments, have broad host ranges, or find themselves in a perpetual co-evolutionary arms race with their host, this is particularly important.

## When redundancy is not redundancy at all

Redundancy is not the only explanation for the absence of phenotypes associated with genetic mutations. Whether a gene is required for pathogenesis can vary depending on the host examined, the conditions under which gene requirements are assessed or the type and sensitivity of the assay used. As a consequence, what may be perceived as redundancy is instead an inability to detect phenotypes using a particular experimental system. For example, the IDTS SdhA is essential for *L. pneumophila* replication in bone marrow-derived primary macrophages but not in cultured U937 cells, a monocyte-derived macrophage cell line (Laguna et al., 2006). Loss of SdhA causes the induction of host cell death in response to *L. pneumophila* challenge (Laguna et al., 2006; Creasey and Isberg, 2012), which is likely to differ between primary and immortalized cells. Similarly, the SidE family of IDTS is important for *L*. *pneumophila* growth in amoebal hosts but is dispensable in primary macrophages (Bardill et al., 2005; Qiu et al., 2016). In these two cases, the host cell type greatly impacts whether a gene is designated as important for *L. pneumophila* pathogenesis. While redundancy is becoming the default justification for a lack of phenotypes, it is not always the culprit. As more effectors and the host processes they modulate are characterized, key differences between effectors that appear to be redundant will most certainly be revealed.

## Methods to resolve redundancy

A significant body of work has focused on defining the role of individual virulence factors in isolation, yet understanding how these components coordinately contribute to pathogenesis is necessary to define key determinants of disease. This is particularly important for pathogens that employ compensatory virulence strategies, as redundancy can greatly impact the ability to define what a pathogen requires to survive and grow within a host. A number of strategies have emerged to address redundancy in bacterial pathogenesis that encompass genetic, biochemical and bioinformatics-based techniques. While many of the approaches do not specifically determine redundant mechanisms at a molecular level, they do define functional relationships between individual proteins, and in some cases the host pathways they target, enabling targeted analyses to decipher the basis of redundancy at multiple levels.

### Brute-force characterization one effector at a time

Combined biochemical, molecular, and cell biological characterization of effectors is the most comprehensive way to identify redundant proteins. It provides detailed information about their mechanism of action, their host cell targets and their direct impact on host cellular processes. Moreover, deciphering the intricate details of an effector's function can define subtle distinctions between effectors with overlapping functions and thus circumvent their improper classification as redundant. However, this method is not without its drawbacks. The amount of time required to exhaustively characterize protein function can be lengthy, especially if the techniques to do so are not available or the function of the host target protein or its component pathway have yet to be characterized. For a pathogen like *L. pneumophila* that employs at least 270 IDTS, such an endeavor would be an arduous one. In addition, for many effectors, sequence homology and structure prediction tools are not always informative. For *L. pneumophila*, as many as one third of all IDTS lack domain homology to any other protein characterized to date and often very little is learned from structural predictions. Finally, while there are several methods to define host targets, including more recently adapted high throughput methods (Mousnier et al., 2014; Yu et al., 2015), this can be challenging as host targets can range from proteins to lipids to small molecules (Machner and Isberg, 2006; Toulabi et al., 2013; Isaac et al., 2015). Thus, while characterizing individual effector functions can be highly informative, the road to defining redundant virulence mechanisms can be bumpy and painstakingly slow.

### Insertional mutagenesis and depletion (iMAD)

iMAD is a genetic screening strategy developed to resolve redundancy amongst effectors by defining sets of bacterial proteins that target common host pathways and parallel pathways exploited by a pathogen to accomplish a single task (O'Connor et al., 2012; O'Connor and Isberg, 2014). To do so, iMAD integrates bacterial mutagenesis and host RNA interference to systematically identify genetic interactions between a pathogen gene and a host gene based on impaired replication of the pathogen (O'Connor et al., 2012; O'Connor and Isberg, 2014). In the case of *L. pneumophila*, a library of transposon mutants were assessed for their ability to replicate within host cells depleted of one of five early secretory proteins that promote *L. pneumophila* intracellular growth (O'Connor et al., 2012). Hierarchical clustering of bacterial gene mutations in IDTS with similar behavioral patterns across all host conditions examined revealed several important functional relationships: (1) Common phenotypic signatures identified sets of bacterial proteins that target common host pathways: these sets of proteins defined distinct functional groups; (2) Deleting pairs of bacterial genes from separate functional groups impaired intracellular growth of *L. pneumophila*: these functional groups defined separate but redundant host pathways targeted by *L. pneumophila* to generate a replication vacuole; (3) Specific defects in host cell biology resulting from loss of bacterial proteins could be predicted for genes based on the characterization of other members of its group: this identified three sets of proteins that independently contribute to the maintenance of replication vacuole membrane integrity; (4) Different combinations of bacterial genes were required for optimal growth in different hosts defining sources of adaptation to host variation. By grouping individual effectors that commonly manipulate a single host pathway and redundant pathways that contribute to a single process, iMAD defines functional relationships between effectors at the target, pathway, cellular process, and system levels. With more efficient methods for generating arrayed bacterial mutant libraries, commercially available RNAi libraries and the development of CRISPR technology to facilitate host cell protein depletion, and the replacement of DNA microarrays with massively parallel sequencing techniques to monitor bacterial mutant populations, more comprehensive, high-throughput iMAD screens are now possible.

### Genome reduction and minimal effector repertoires

Genome reduction followed by effector repertoire reconstitution is another strategy used to identify redundant effectors and the host pathways they target (Cunnac et al., 2011). Progressive removal of all 28 effector genes from the plant pathogen *Pseudomonas syringae Pto* DC3000 determined 15 of the effector genes are collectively dispensable for growth in the plant host *Nicotiana benthamiana* (Kvitko et al., 2009). The subsequent reintroduction of different combinations of effectors defined several redundant-effector groups (REGs) that promote *P*. *syringae* growth in *N*. *benthamiana*, two of which were determined to mediate resistance to independent arms of plant innate immunity (Block and Alfano, 2011; Cunnac et al., 2011). By analyzing correlates between effector combinations and rescued *P*. *syringae* growth during infection, a minimal set of 8 effectors was defined that was sufficient to promote growth of *P*. *syringae* to near wild type levels. The effector repertoire reconstitution linked individual REGs with the host pathways they target, identified specific combinations of effectors that are sufficient to cause disease and demonstrated the ability to swap different members of individual REGs and still achieve robust *P. syringae* growth. For genetically amenable pathogens with manageable sizes of effector repertoires, preferably clustered in a minimal set of genetic loci to facilitate combinatorial effector reintroduction into the genome, effector repertoire reduction, and reconstitution strategies provides a comprehensive method to define redundant effectors, the cellular processes they modulate and the minimum set of effector functions required for pathogenesis.

### Effector interactome mapping

Proteome-based analyses that use high throughput mass spectrometry allow the interactomes of entire effector subfamilies to be mapped. Affinity purified-mass spectrometry has been used to define the host interacting partners of 58 secreted virulence factors in *Chlamydia trachomatis* called inclusion membrane proteins (Incs) (Mirrashidi et al., 2015). The results not only allowed groups of effectors to be assigned to specific cellular processes but identified sets of Inc proteins that target the same host proteins or different members of the same multiprotein complex. Mapping the interactome network of all 58 Inc proteins revealed sets of Inc proteins that converge on common targets, pathways and cellular processes: this defined focal points of host modification by *C. trachomatis* and thus, potential sources of redundancy. Moreover, the *C. trachomatis* Inc-human interactome had significant overlap with that of other pathogens. Comparisons with three viral-human interactomes (Jager et al., 2011; Davis et al., 2015; Ramage et al., 2015) identified 98 shared host targets between *C. trachomatis* and at least one of the three viruses. Similarly, a number of the Inc host protein targets are also common targets of *L. pneumophila* IDTS including Rtn4 (Kotewicz et al., 2016), vATPases (Xu et al., 2010), and the retromer complex (Finsel et al., 2013). The lack of similarly between the respective *C. trachomatis* Inc proteins and *L. pneumophila* IDTS and differences in the host protein complex subunits targeted demonstrates that each bacterial pathogen has acquired or evolved different mechanisms to modulate the same host proteins, pathways and/or cellular processes. Comparing effector functions between pathogens not only allows additional redundant virulence mechanisms to be defined but establishes a critical set of events central to microbial pathogenesis.

### Comparative and functional genomics can predict redundant virulence mechanisms

**Figure 2:**
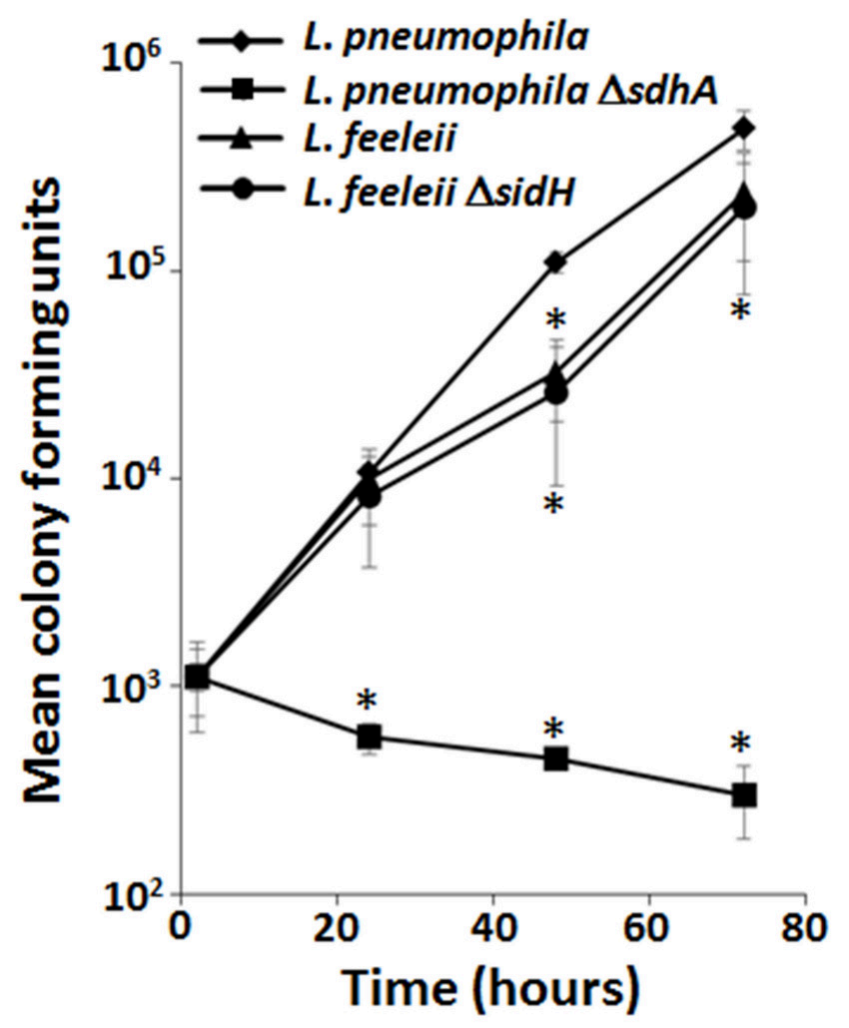
Lack of the SidH family of Dot/Icm translocated substrates does not impair growth of *L. feeleii* in macrophages despite being indispensable in *L. pneumophila*. Growth of wild type *L. pneumophila, L. pneumophila* Δ*sdhA*, wild type *L*. *feeleii* and *L*. *feeleii* Δ*sidH* in A/J mouse bone marrow-derived macrophages, based on recovered colony forming units (CFU) on solid media from lysed host cells, was monitored over 72 h encompassing 3 consecutive rounds of infection (Supplemental Material). Plotted is the total bacterial yield at the indicated time points normalized to the *L. pneumophila* wild-type strain by the number of intracellular bacteria 2 h post infection. Data are representative of at least 2 independent experiments ± standard deviation of 3 replicates. An asterisk indicates a *P* < 0.05 based on a Student's *t*-test relative to the *L. pneumophila* wild type strain.

Comparative genomics combined with phenotypic analyses provide a means to define correlates between effector conservation and redundant virulence mechanisms (Baltrus et al., 2011). The *P*. *syringae* pan-genome effector repertoire consists of 57 effectors but different subsets of effectors are sufficient for growth in the same plant host. Computational analyses that correlate specific combinations of effectors and host tropism (Baltrus et al., 2011) allow redundant virulence mechanisms to be elucidated on a global scale. Alternatively, comparative genomes can be used as a more targeted approach. For example, in *L. pneumophila* SdhA is a critical virulence determinant in macrophages (Laguna et al., 2006). While the precise function of SdhA is still unclear, the severe growth defect of the Δ*sdhA* mutant is due to loss of vacuole integrity that leads to a robust host innate immune response and consequently either bacterial or host cell death (Laguna et al., 2006; Creasey and Isberg, 2012). *L. pneumophila* encodes two paralogs of SdhA, SidH, and SdhB, but their deletion only moderately enhances the already severe intracellular growth defect of the Δ*sdhA* mutant (Laguna et al., 2006). *Legionella feeleii* lacks a *sdhA* paralog but grows almost as well as the wild type strain of *L. pneumophila* in macrophages (Figure 2). While the presence of *sidH* in *L. feeleii* may compensate for the absence of *sdhA*, deletion of *sidH* does not impair *L. feeleii* growth in macrophages (Figure 2). The dispensability of SdhA (and SidH) in *L. feeleii* is not due to the lack of *plaA* and/or *traI*, which suppresses the Δ*sdhA* mutant phenotype in *L. pneumophila* (Creasey and Isberg, 2012). Thus, while SdhA plays a critical role in *L. pneumophila* pathogenesis, the entire family of SidH paralogs is dispensable in *L. feeleii*. While there are a number of explanations for this discrepancy, *L. feeleii* encodes 27 additional putative IDTS that are not conserved in *L. pneumophila* (Burstein et al., 2016), one or more of which may compensate for the absence of SdhA despite their lack of homology. As more genomes of pathogen isolates are sequenced, correlates between effector conservation and phenotypes will allow alternate virulence mechanisms employed by pathogens to be defined.

## Future directions

Much of the research in microbial pathogenesis employs a reductionist's approach, where the individual components are investigated in isolation. While this strategy has proven extremely useful in identifying key players and their functions, it does not offer tremendous insight into the complex interactions that exist at the systems level. Pathogens invest an incredible amount of resources to build a robust virulence strategy. Redundancy allows pathogens to rapidly adapt to frequently changing environments and the elaborate, multi-tiered antimicrobial strategies employed by their hosts. As more and more effectors are characterized, a striking pattern of redundancy is beginning to emerge. In this review, we establish a structured nomenclature for the different forms of redundancy observed across multiple levels of biological organization. The types of redundancy defined here are not mutually exclusive nor are they expected to be exhaustive as more virulence factors are characterized. Instead, we offer a framework to generate a broader, more dynamic view of the mechanisms governing microbial pathogenesis.

## Author contributions

SG generated data presented in the manuscript. SG and TO wrote the manuscript.

### Conflict of interest statement

The authors declare that the research was conducted in the absence of any commercial or financial relationships that could be construed as a potential conflict of interest.
