# Supplementary material for: Beyond Paralogs: The Multiple Layers of Redundancy in Bacterial Pathogenesis
Source: Front Cell Infect Microbiol. 2017 Nov 15;7:467. doi: 10.3389/fcimb.2017.00467 (PMC5694747; doi:10.3389/fcimb.2017.00467)
Supplement: Supplementary file 1 [file DataSheet1.pdf]

Supplemental Material

Beyond Paralogs: The Multiple Layers of Redundancy in Bacterial Pathogenesis

Soma Ghosh[1] and Tamara J. O'Connor[1*]

[1]Department of Biological Chemistry, Johns Hopkins University School of Medicine, Baltimore, MD, USA

***Correspondence:**
Tamara J. O'Connor
toconno7@jhmi.edu

1 Supplementary Data

Materials and Methods

Bacterial strains, cultured cells and growth media. All *Legionella* strains were generated in the *Legionella pneumophila* Philadelphia-1 strain Lp02 (Berger and Isberg, 1993) thy+ revertant strain (O'Connor *et al*., 2011) or *Legionella feeleii* strain WO-44C (American Tissue Type Collection, ATCC35072) (Herwaldt *et al*., 1984). *Legionella* strains were cultured at 37°C in liquid *N*-(2-acetamido)-2-aminoethanesulfonic acid (ACES) buffered yeast extract (AYE) media or on solid charcoal ACES-buffered yeast extract (CYE) media (Feeley *et al*., 1979; Gabay and Horwitz, 1985) supplemented with L-cysteine sulfate (0.4 mg/ml) and ferric nitrate (0.135 mg/ml) and, when appropriate, 40 μg/ml kanamycin or 5% sucrose. Plasmids were introduced into *Legionella* by electroporation (Berger *et al*., 1994). The *E. coli* strain DH5α λpir was used for all plasmid cloning. *E. coli* strains were grown in liquid Luria broth (LB) or on solid LB plates supplemented with 50 μg/ml kanamycin when appropriate. All bacterial strains, plasmids and primers are summarized in Table S1. Primary bone marrow-derived macrophages from A/J mice were isolated and cultured as previously described (Berger and Isberg, 1993).

Construction of *Legionella* deletion mutants. Null mutations in individual genes were generated in using a double recombination strategy employing the suicide vector pSR47s as previously described (Merriam *et al*., 1997). Primer pairs for plasmid construction are listed in Table S1. All plasmids were sequenced prior to use. For each mutant, 12-16 individual isolates were screened by the polymerase chain reaction (PCR) and intracellular growth phenotypes of at least two independent isolates were compared.

Intracellular Growth Assays

Intracellular growth assays of *L. pneumophila* in A/J macrophages was performed as described in elsewhere (Berger and Isberg, 1993).

Ethics Statement

This study was carried out in accordance with the recommendations of and protocol approval of the Johns Hopkins Institutional Biosafety Committee.

2 Supplementary Tables

Table S1: Strain, plasmids and oligonucleotides

Strains

Strain	Genotype	Description	Reference
L. pneumophila			
TO1249	Philadelphia-1, *rpsL*$^{-}$, *hsdR*$^{-}$, *thyA*$^{+}$	wild type strain	Berger and Isberg, 1993
TO886	Δ*lpg0376*	*sdhA* deletion strain	Laguna *et al*., 2006
L. feeleii			
TO1632		wild type strain (ATCC 35072)	Herwaldt *et al*., 1984
TO2311	Δ*lf0633*	*sidH* homolog (*lf0633*) deletion strain	this work

Plasmids

Plasmids	Features	Description	Reference
pSR47s	R6K, *sacB*, Km^{R}	*Legionella* suicide vector	Merriam *et al*., 1997
pTO936	*lf0633* flanking regions, Km^{R}, *sacB*,	*lf0633* deletion plasmid	this work

***Oligonucleotides*[1]**

Name	Sequence[1]
lf0633up1	CGTAGAGCTCCTTTCCAATTGCCGTGAATT
lf0633up2	CGTAGGATCCTTTTCAATCCAGTCACTTTA
lf0633dn1	CGTAGGATCCCCAATAATTACGTTCTGAAA
lf0633dn2	CGTAGTCGACTGTGTCAGTGCCAGGCTTTT

[1] Restriction enzyme sites are underlined

3 References

Berger, K. H., Merriam, J. J., Isberg, R. R. (1994). Altered intracellular targeting properties associated with mutations in the *Legionella pneumophila dotA* gene. *Mol Microbiol* 14, 809-822.

Berger, K. H., Isberg, R. R. (1993). Two distinct defects in intracellular growth complemented by a single genetic locus in *Legionella pneumophila*. *Mol Microbiol* 7, 7-19.

Feeley, J. C., Gibson, R. J., Gorman, G. W., Langford, N. C., Rasheed, J. K., Mackel, D. C., Baine, W. B. (1979). Charcoal-yeast extract agar: primary isolation medium for *Legionella pneumophila*. *J Clin Microbiol* 10, 437-441.

Gabay, J. E., Horwitz, M. A. (1985). Isolation and characterization of the cytoplasmic and outer membranes of the Legionnaires' disease bacterium (*Legionella pneumophila*). *J Exp Med* 161, 409-422.

Herwaldt, L. A., Gorman, G. W., McGrath, T., Toma, S., Brake, B., Hightower, A. W., *et al*., (1984). A new *Legionella* species, *Legionella feeleii* species nova, causes Pontiac fever in an automobile plant. *Ann Intern Med* 100, 333-338.

Laguna, R. K., Creasey, E. A., Li, Z., Valtz, N., and Isberg, R. R. (2006). A *Legionella pneumophila*-translocated substrate that is required for growth within macrophages and protection from host cell death. *Proc Natl Acad Sci* USA 103, 18745–18750. doi: 10.1073/pnas.0609012103

Merriam, J. J., Mathur, R., Maxfield-Boumil, R., Isberg, R. R. (1997). Analysis of the *Legionella pneumophila fliI* gene: intracellular growth of a defined mutant defective for flagellum biosynthesis. *Infect Immun* 65, 2497-2501.

O'Connor, T. J., Adepoju, Y., Boyd, D., Isberg, R. R. (2011). Minimization of the *Legionella pneumophila* genome reveals chromosomal regions involved in host range expansion. *Proc Nat Acad Sci USA* 108, 14733-14740.
